# Optimizing SGLT2 inhibitor and GLP-1 RA prescribing in high-risk patients with diabetes: a Department of Veterans Affairs quality improvement intervention

**DOI:** 10.1186/s12875-025-02709-0

**Published:** 2025-03-21

**Authors:** Shira Yun, Kathryn Hurren, Rob Holleman, Mandi Klamerus, Adam Tremblay, Jeremy B. Sussman

**Affiliations:** 1 VA Ann Arbor Healthcare System, Ann Arbor, MI USA; 2 VA Center for Clinical Management Research, Ann Arbor, MI USA; 3 Department of Internal Medicine, University of Michigan, Ann Arbor, MI USA; 4 Institute for Healthcare Policy and Innovation, Ann Arbor, MI USA; 5 2800 Plymouth Road, Building 16, Room 335E, Ann Arbor, USA

**Keywords:** Sodium glucose cotransporter-2 (SGLT2) inhibitor, Glucagon-like peptide-1 receptor agonist (GLP-1RA), Veterans, Quality improvement, Type 2 diabetes, Medication therapy management

## Abstract

**Introduction:**

Glucagon-like peptide-1 receptor agonists (GLP-1 RA) and sodium glucose cotransporter-2 (SGLT2) inhibitors have dramatic clinical benefits, but many appropriate patients do not receive them. We developed a quality improvement (QI) intervention to increase the adoption of these drugs in patients with type 2 diabetes (T2D) and atherosclerotic cardiovascular disease (ASCVD), chronic kidney disease (CKD), and/or heart failure (HF). The purpose of this study was to examine whether the intervention increased the use of SGLT2 inhibitors and GLP-1 RAs.

**Methods:**

The intervention included: (1) education, academic detailing (1:1 pharmacist to clinician coaching), and audit and feedback directed at providers and allied health professionals at the Veterans Affairs Ann Arbor Healthcare System (VAAAHS); (2) outreach and inreach to patients with T2D and ASCVD, CKD, and/or HF who were not on GLP-1 RAs or SGLT2 inhibitors at baseline. Patients were identified and outcomes evaluated using existing VA national reports. We performed a difference-in-difference analysis of the change in GLP-1 RA and SGLT2 inhibitor prescribing rates before, during, and after the intervention, comparing rates in VAAAHS to rates in the same VA region (called a Veterans Integrated Service Network (VISN)) and the VA nationally to determine whether the rates of prescribing increased faster in VAAAHS than the VISN or VA nationally.

**Results:**

Home telehealth nurses and clinical pharmacy practitioners (CPPs) provided outreach to 445 patients; 48% (*n* = 215) of whom initiated SGLT2 inhibitors or GLP-1 RAs. Four CPPs provided 101 academic detailing sessions to 72 providers. Prior to the intervention, the prescribing rate was 22.7% in VAAAHS, 20.3% in the VISN 10 region, and 18.7% in VA nationally. At the end of the 12-month intervention, the prescribing rate had increased to 37.9% in VAAAHS, 28.4% in the VISN 10 region, and 26.5% in VA nationally. Six-months post-intervention, the prescribing rate continued to increase to 42.4% in VAAAHS, 32.2% in the VISN 10 region, and 30.2% in VA nationally. The rate of prescribing growth in VAAAHS was significantly faster than in the VISN or VA nationally (*p* < 0.001).

**Conclusion:**

Our multidisciplinary QI intervention increased SGLT2 inhibitor and GLP-1 RA prescribing approximately 8% points faster than the national average.

**Supplementary Information:**

The online version contains supplementary material available at 10.1186/s12875-025-02709-0.

## Introduction

Among the most important changes in diabetes management in the last generation is the dramatic clinical benefits produced by select glucagon-like peptide-1 receptor agonists (GLP-1 RA) and sodium glucose cotransporter-2 (SGLT2) inhibitors. Select agents from both classes reduce major adverse cardiovascular events (MACE) and improve kidney outcomes, and SGLT2 inhibitors additionally reduce heart failure (HF) hospitalizations in patients with type 2 diabetes (T2D) with a history of or at high risk for atherosclerotic cardiovascular disease (ASCVD), and there are similar benefits in other populations [1–3]. The American Diabetes Association (ADA) recommends these medications in patients with T2D and ASCVD, chronic kidney disease (CKD), and/or HF, independent of glycemic control or metformin use [1]. 

Despite their remarkable benefits, these medications are underused [4, 5]. Our internal data showed only a fraction of patients with both diabetes and ASCVD, CKD, and/or HF were prescribed either of these medication classes. What is less clear is why and how this can be remedied. Within the U.S. Department of Veterans Affairs (VA), including our facility, we believe that limited prescribing uptake of SGLT2 inhibitors and GLP-1 RAs was due to a lack of prescriber experience with these medication classes, discomfort with adjusting diabetes regimens in patients who are already meeting targets, and, in the past, a belief that their use was not supported internally by VA [5]. For example, until February 2021, the sole SGLT2 inhibitor available (empagliflozin) was available only after meeting certain criteria, and the GLP-1 RA semaglutide required prior authorization from a pharmacist. Despite this, the VA has many resources that could speed up adoption, including a national information technology architecture with reports to guide appropriate prescribing and universal multidisciplinary Patient Aligned Care Teams (PACTs) for all outpatient primary care. We hypothesized barriers to appropriate uptake of SGLT2 inhibitors and GLP-1 RAs were addressable and a substantial increase in appropriate use would improve patient care and, ultimately, patient outcomes.

Given this opportunity, we developed a quality improvement (QI) intervention to aid the adoption of SGLT2 inhibitors and GLP-1 RAs in patients with T2D and ASCVD, CKD, and/or HF. Our intervention used existing clinical reports and a limited amount of nurse and clinical pharmacy practitioner (CPP) time (in addition to regular scheduled patient care). Here, we describe this intervention and evaluate its impacts on the prescribing of SGLT2 inhibitors and GLP-1 RAs. (Of note, our study did not address potential overuse of these medicines.)

## Methods

This project was approved as a quality improvement (non-research) project by the VA Ann Arbor Healthcare System (VAAAHS) Quality Management Department. The primary analysis would compare rates of use of SGLT2 inhibitors and GLP-1 RAs in patients with T2D and ASCVD, CKD, and/or HF in VAAAHS to all patients in the region that includes Ann Arbor (i.e., Veterans Integrated Service Network [VISN] 10) and all patients in VA nationally over time.

### Setting and participants

This intervention was conducted at VAAAHS from October 1, 2021, to September 30, 2022. VAAAHS is comprised of the LTC Charles S. Kettles VA Medical Center, an academic teaching hospital affiliated with the University of Michigan, as well as several community-based outpatient clinics (CBOCs). VAAAHS primary care is organized in PACTs, which includes primary care clinicians, licensed practical nurses, and registered nurses. A CPP supports one PACT, providing care for 3,600 patients on average with 80% of their schedule bookable for chronic disease management in addition to PACT support. CPPs are licensed independent providers with prescriptive authority in the VA healthcare system. Home telehealth nurses support chronic disease state management in PACT patients enrolled in their program by monitoring transmitted patient data (e.g., glucose, blood pressure, weight), providing education, and facilitating medication adjustments by CPPs or primary care providers (PCPs).

The VA Pharmacy Benefits Management (PBM) Academic Detailing Services (ADS) Diabetes Informatics Toolset includes both the Patient Report and Trend Report. The Patient Report was used to identify patients with T2D and comorbid ASCVD, CKD, and/or HF who may benefit from SGLT2 inhibitor or GLP-1 RA therapy. The Trend Report was used to evaluate prescribing trends at the facility, region, and national level. These existing reports, which pull information from the VA’s Corporate Data Warehouse (CDW), are continuously updated.

Eligibility for inclusion on the Patient Report and Trend Report was defined nationally in the Diabetes Information Toolset, not by the authors. The Patient Report is updated daily and defines having diagnosis of T2D by ICD-9/10 code or a most recent A1c ≥ 7%. ASCVD and HF are defined by ICD-9/10 code, and CKD is defined as an estimated glomerular filtration rate (eGFR) of 25–59 without dialysis. Only active VA patients are included in the Patient Report, defined as patients having no date of death and any of the following: (1) an outpatient visit within the past year, (2) a future appointment scheduled, (3) a primary provider assigned, and/or (4) any active prescription. Patients with diagnosis codes for type 1 diabetes, pancreatic cancer, or pancreatitis are not included in the Toolset reports. The Patient Report includes patients without baseline prescription for or documented allergy to any SGLT2 inhibitor or GLP-1 RA. Initially, patients were required to be prescribed or have a documented adverse drug event to metformin to be included in the reports. On July 21, 2022, this criterion was removed from the Toolset for all patient cohorts except patients with T2D and ASCVD. Inclusion in the Patient Report determined eligibility for the QI intervention. The Trend Report provides data quarterly, including the percentage of patients prescribed a SGLT2 inhibitor and/or GLP-1 RA from all active patients identified as having T2D and ASCVD, CKD, and/or HF who would potentially benefit from these medications as described above.

The two comparison groups were all patients in the region that includes Ann Arbor (i.e., Veterans Integrated Service Network [VISN] 10) and all patients in VA nationally. The VISN 10 region is comprised of Michigan, Indiana, and Ohio. Thus, both comparison groups included VAAAHS, though it was only a small percentage of the total number of patients.

### Data sources

Information for the outcomes came from the national Diabetes Informatics Toolset, ensuring consistency in data definitions for comparison among facility (VHAAHS), region (VISN 10), and national cohorts over time. The number of patients who received outreach was obtained by examining note titles from the EHR.

### Intervention description

**Fig. 1:**
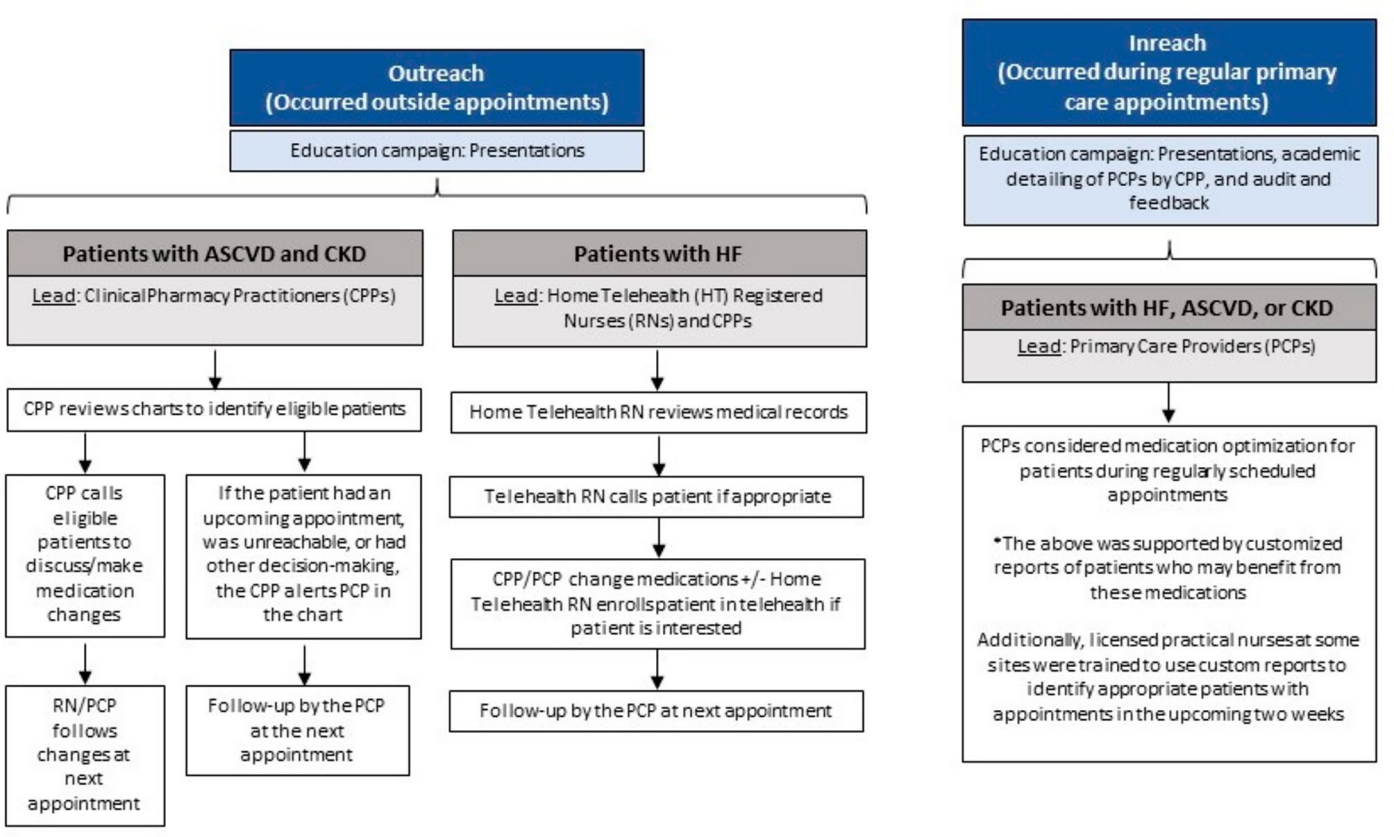
Overview of the quality improvement intervention. ASCVD = atherosclerotic cardiovascular disease, CKD = chronic kidney disease, CPP = clinical pharmacy practitioner, HF = heart failure, HT = Home Teleheath, PCP = primary care provider, RN = registered nurse

The QI intervention had two components: (1) an education campaign to increase knowledge and awareness of newer evidence-based diabetes management; and (2) a diabetes medication optimization program that included outreach (defined as unscheduled contact with the patient outside an appointment) and inreach (defined as an intervention at the time of a scheduled appointment) (Fig. 1). Outreach and inreach efforts were concurrent and all identified patients were eligible for both/either opportunity to optimize diabetes therapy. The outreach effort was limited by clinician time constraints as there were no dedicated personnel for this “real world” intervention. Initiation of SGLT2 inhibitors was preferred over GLP-1 RAs when appropriate due to stronger evidence for benefit in CKD and HF as well as formulary considerations.

Initially, the educational campaign consisted of six presentations (delivered in September and October of 2021) to describe the evidence for SGLT2 inhibitor and GLP-1 RA use, share current guidelines/recommendations, define contraindications, and issue a call to action. Presentations were led by a certified diabetes care and education specialist CPP and a PCP. Presentations were tailored for the recipients, which included separate sessions with PCPs, nurses, and pharmacists. Additionally, targeted one-on-one academic detailing educational sessions to review information, discuss implementation, and view individualized Patient Reports were conducted by CPPs throughout the intervention to support this educational effort.

CPPs and home telehealth RNs who participated in the outreach effort did so in addition to their regular scheduled patient care; there were no dedicated personnel or time for this project as this was an unfunded QI initiative. The outreach effort was divided into two clinical cohorts based on clinical indication. Patients with T2D and ASCVD and/or CKD were evaluated by CPPs with prescriptive authority. CPPs reviewed patient records, documented their clinical findings with a templated note in the EHR, and called eligible patients to provide education and discuss initiation of a SGLT2 inhibitor or GLP-1 RA, adjust other medications, and schedule follow-up. Alternatively, if the patient had an upcoming PCP appointment, could not be reached, or required additional clinical decision-making, CPPs made recommendations and alerted PCPs without making medication changes. Patients with T2D and HF with or without ASCVD or CKD, who require additional clinical monitoring and adjustments for factors such as volume status and blood pressure management, received more frequent monitoring via home telehealth. The medical records of patients with T2D and HF were reviewed by home telehealth nurses. The nurse called the patient to provide education and discuss the clinical benefits of adding a SGLT2 inhibitor to his/her medication regimen. These patients were offered the opportunity to enroll in a home telehealth program for blood glucose, blood pressure, and weight monitoring surrounding the medication change, including collaborative adjustment of other glucose-lowering, antihypertensive, and diuretic medications. Clinical decision making and prescribing were done in collaboration with the CPP or PCP.

In our inreach effort, which was concurrent to the outreach effort, PCPs were encouraged to optimize drug regimens with a SGLT2 inhibitor or GLP-1 RA for all appropriate patients during regularly scheduled primary care appointments. This effort was supported by providing a customized report to providers of patients on their panel who may benefit from these medications and monthly audit and feedback on clinic-wide prescribing trends at meetings. Additionally, licensed practical nurses at some sites were trained to use custom Patient Reports to identify patients eligible for SGLT2 inhibitor or GLP-1 RA who were scheduled for appointments in the upcoming two weeks and to alert the PCP to consider medication optimization at the time of the appointment.

### Evaluation

We obtained data from July 2020 to March 2023, providing 15 months of data before the year-long intervention began and six months afterwards. We used the RE-AIM implementation framework to guide the evaluation [6, 7]. The RE-AIM Framework consists of five dimensions: reach into the target population; effectiveness of the intervention; adoption by target settings, institutions and/or staff; implementation, including consistency of delivery; and maintenance of intervention effects over time.

We report GLP-1 RA and SGLT2 inhibitor prescribing rates before, during, and after the intervention, comparing rates in VAAAHS to rates in VISN 10 and the VA nationally. A difference-in-difference analysis was conducted to determine whether the rates of prescribing increased faster in VAAAHS compared to the VISN 10 region and VA nationally using logistic regression. The dichotomous outcome variable was whether a SGLT2 inhibitor or GLP-1 RA was prescribed based on data from the Trend Report. The quarters were categorized as pre, during, and post intervention. Pre intervention was July 2020-September 2021, during intervention was October 2021-September 2022, and post intervention October 2022-March 2023. VAAAHS, the VISN 10 region, and VA nationally were included as a categorical site variable. The model included an interaction between intervention period and site. Postestimation predicted probabilities were obtained from the model. All analyses were performed using Stata 17.0.

## Results

**Fig. 2:**
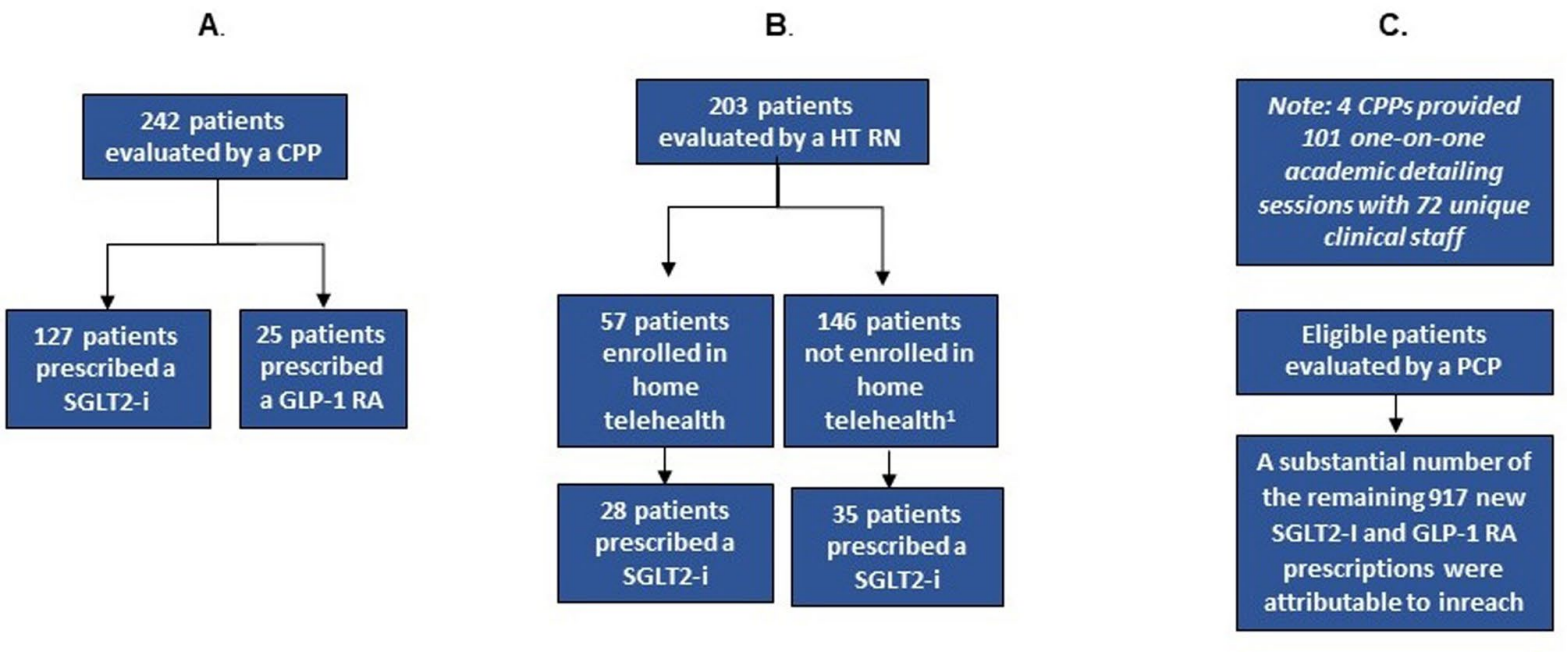
(**A**) Outreach performed by Clinical Pharmacy Practitioners. CPP = clinical pharmacy practitioner, GLP-1 RA = glucagon-like peptide-1 receptor agonist, SGLT2-i = sodium glucose cotransporter-2 inhibitor. (**B**) Outreach performed by Home Telehealth Registered Nurses. Many patients were found to be not eligible for enrollment (e.g., homeless, could not be reached). HT RN = home telehealth registered nurse, SGLT2-i = sodium glucose cotransporter-2 inhibitor. (**C**) Inreach performed by primary care providers. CPP = clinical pharmacy practitioner, GLP-1 RA = glucagon-like peptide-1 receptor agonist, PCP = primary care provider, SGLT2-i = sodium glucose cotransporter-2 inhibitor

At the beginning of the intervention, eligible patients with T2D and ASCVD, CKD, and/or HF were identified by the Patient Report as potentially benefiting from SGLT2 inhibitor or GLP-1 RA initiation. CPPs and home telehealth nurses provided outreach to as many patients as possible, while continuing to complete their regular scheduled clinical duties. During the intervention period, they were able to provide outreach to a total of 445 patients (Fig. 2A and B). Of the patients with T2D and ASCVD or CKD, CPPs evaluated 242. Of these patients, 63% (*n* = 152) were prescribed a SGLT2 inhibitor (*n* = 127) or GLP-1 RA (*n* = 25) (Fig. 2A). Home telehealth nurses provided outreach to 203 patients with T2D and HF. Of these, 28% (*n* = 57) were newly enrolled in-home telehealth as part of the outreach and 49% (*n* = 28) of those enrolled were prescribed a SGLT2 inhibitor. Of the 72% (*n* = 146) of patients who were not enrolled in home telehealth, 24% (*n* = 35) were prescribed a SGLT2 inhibitor (Fig. 2B). For the inreach effort, 4 CPPs provided one-on-one academic detailing regarding SGLT2 inhibitors and/or GLP-1RAs in 101 sessions with 72 unique clinical staff during the intervention. Eligible patients were evaluated by a PCP for medication optimization at routine scheduled appointments (Fig. 2C). (The results are described in terms of the RE-AIM Framework in Additional File 1 [6]). 

**Fig. 3:**
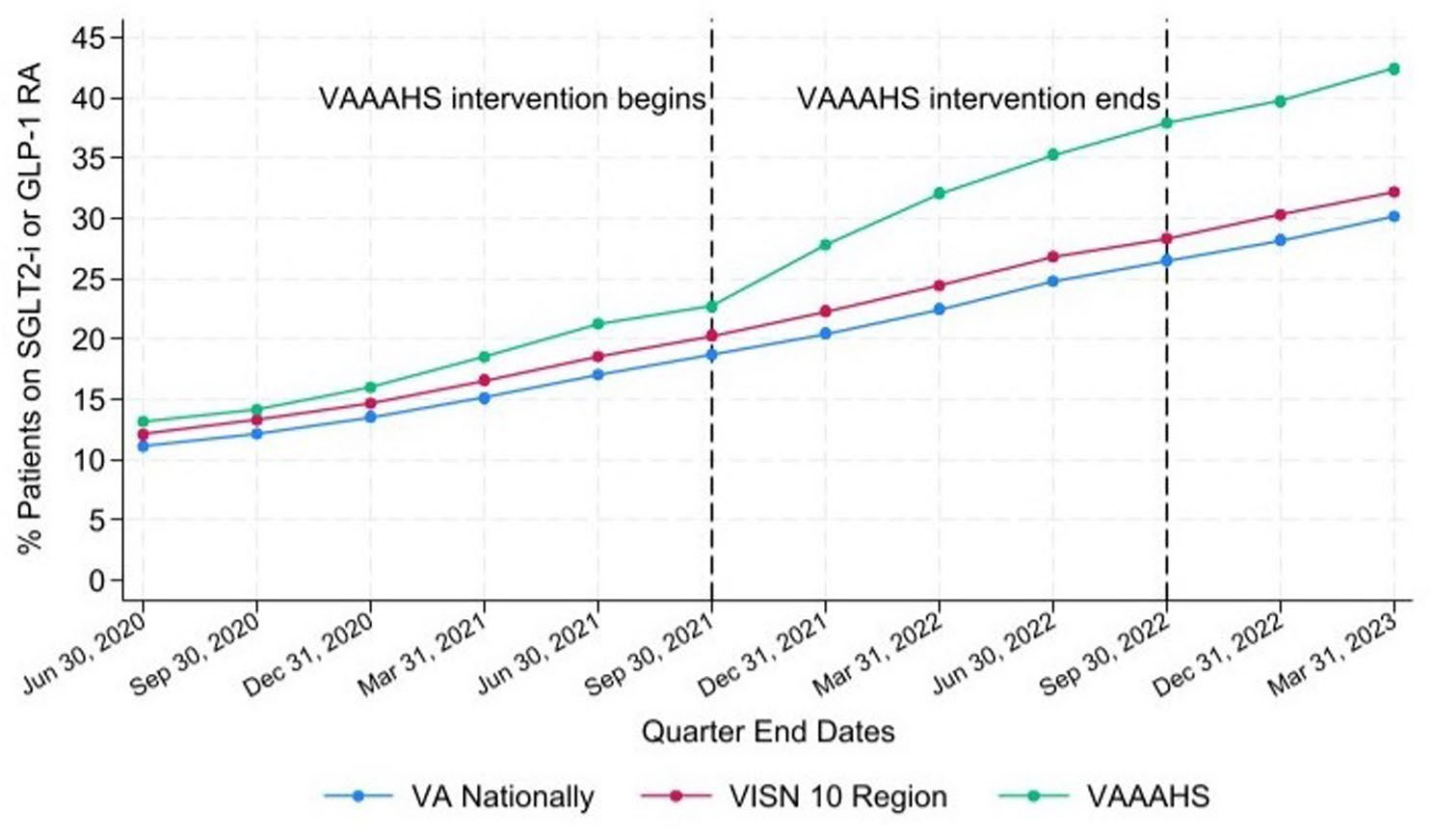
Percentage of patients with T2DM and CKD, ASCVD, or HF on a GLP1-RA or SGLT2-i. ASCVD = atherosclerotic cardiovascular disease, CKD = chronic kidney disease, GLP1-RA = glucagon-like peptide-1 receptor agonist, HF = heart failure, SGLT2-i = sodium glucose cotransporter-2 inhibitor, T2DM = Type 2 diabetes mellitus, VAAAHS = Veterans Affairs Ann Arbor Healthcare System, VISN = Veterans Integrated Service Network

Prior to the intervention, the prescribing rate was 22.7% (1,665/7,336) in VAAAHS, 20.3% (13,068/64,337) in the VISN 10 region, and 18.7% (126,822/676,814) in VA nationally (Table 1; Fig. 3). At the end of the 12-month intervention, the prescribing rate increased to 37.9% (2,797/7,374) in VAAAHS, 28.4% (16,605/58,525) in the VISN 10 region, and 26.5% (173,837/654,994) in VA nationally. Six-months post-intervention, the prescribing rate continued to increase to 42.4% (3,086/7,275) in VAAAHS, 32.2% (18,707/58,041) in the VISN 10 region, and 30.2% (198,553/657,365) in VA nationally. The increases in prescribing rates over time at VAAAHS, the VISN 10 region, and VA nationally were all statistically significant and the increases in VAAAHS were significantly faster (*p* < 0.001) (Additional File 2).

**Table 1:** Patients with type 2 diabetes and chronic kidney disease, atherosclerotic cardiovascular disease, or heart failure on a sodium glucose cotransporter-2 inhibitor or glucagon-like peptide-1 receptor agonist [Information obtained from the Trend Report]

Location	Value	Pre-Intervention						Intervention				Post-Intervention	
		June 30, 2020	Sept 30, 2020	Dec 31, 2020	March 31, 2021	June 30, 2021	Sept 30, 2021	Dec 31, 2021	March 31, 2022	June 30, 2022	Sept 30, 2022	Dec 31, 2022	March 31, 2023
VA nationally	Numerator	77,516	84,816	93,677	103,656	115,974	126,822	137,813	150,148	164,212	173,837	185,080	198,553
	Denominator	697,150	696,619	690,700	685,058	678,768	676,814	672,355	667,128	661,420	645,994	655,498	657,365
	Percentage	11.1	12.2	13.6	15.1	17.1	18.7	20.5	22.5	24.8	26.5	28.2	30.2
VISN 10 region	Numerator	8,056	8,864	9,663	10,806	11,996	13,068	14,248	15,417	16,616	16,605	17,606	18,707
	Denominator	66,525	66,614	65,726	64,985	64,594	64,337	63,839	63,035	61,847	58,525	58,061	58,041
	Percentage	12.1	13.3	14.7	16.6	18.6	20.3	22.3	24.5	26.9	28.4	30.3	32.2
VAAAHS	Numerator	934	1,048	1,148	1,347	1,549	1,665	2,038	2,308	2,603	2,797	2,883	3,086
	Denominator	7,081	7,394	7,169	7,267	7,272	7,336	7,318	7,191	7,369	7,374	7,258	7,275
	Percentage	13.2	14.2	16	18.5	21.3	22.7	27.9	32.1	35.3	37.9	39.7	42.4

Numerator: Number of Patients on SGLT2-i or GLP1-RA

Denominator: Number of Patients with T2DM with CKD, ASCVD, or HF

VAAAHS = Veterans Affairs Ann Arbor Healthcare System, VISN = Veterans Integrated Service Network

There were 1,665 patients on recommended SGLT2 inhibitors or GLP-1 RAs at the start of the intervention, whereas at the end of the intervention period 2,797 patients were on these medications. We observed 215 patients were prescribed an SGLT2- or GLP-1 RA due to the outreach arm (Fig. 2A and B). Although not precisely quantifiable given a non-static cohort, a substantial number of the remaining 917 new prescriptions were likely attributable to the inreach intervention (Fig. 2C).

Clinician participation in the outreach effort included 100% of home telehealth nurses (*n* = 7) and 81% of CPPs (13 of 16) who were invited to participate. Post-intervention, participating telehealth nurses and CPPs received a brief survey containing seven questions. The overall response rate was 50%, including responses from 6 (46%) CPPs and 4 (57%) nurses (Additional File 1). Respondents varied in the level of intervention support they provided with 33% reaching out to greater than 20 patients, 22% reaching out to 11–20 patients, and 44% reaching out to 10 or less patients. Respondents spent on average 17 min at each outreach encounter. Respondents indicated that the following items facilitated their outreach efforts: education and communication about the purpose of the project and guidelines for qualifying for medication optimization, templates to assist with documentation and review, strong communication among team members, and reports that were easy to use. Barriers to outreach efforts included a lack of time to complete outreach and difficulty reaching patients.

## Discussion

We developed a multidisciplinary, multifaceted intervention that significantly improved prescribing rates of SGLT2 inhibitors and GLP-1 RAs, medications that have been shown to reduce MACE, slow the progression of kidney disease, and improve outcomes in heart failure. Our intervention used an existing, national Diabetes Informatics Toolset and regular clinical staff support, with no other technology or dedicated personnel. The intervention appears to have been successful. We found a greater increase in use in intervention sites than in other areas across the country, despite rapid national growth in use.

This work has clear practice implications. Increasing the speed of adoption for healthcare innovation is a key concern for healthcare research and practice [8, 9]. The medications in the QI intervention have excellent randomized trial evidence supporting their use, have relatively few side effects, and recently had formulary restrictions reduced. Clinical practice guidelines from multiple organizations changed to more strongly encourage their use just before and during the intervention [1, 10, 11]. We found that the primary barriers were gaps in knowledge and in becoming accustomed to consistent use of the medicines. We addressed these with our intervention, which provided information on how to use the medications, had clinical leadership actively encouraging their use, and we created audit and feedback reports about progress on prescribing trends. Given the great impact these medications have, increasing the speed of adoption with QI is a relevant tool that could be used by many clinics and health systems nationwide.

Both our outreach and inreach interventions were effective. The success of our outreach efforts fits with other research on the benefits of pharmacist engagement in primary care [12, 13]. The inreach program, which consisted of education, academic detailing, audit and feedback and customized reports, influenced normative prescribing at routine primary care appointments. The success of the inreach effort was attributable to the wholescale level of engagement across VAAAHS and resulted in the majority of prescribing during the intervention.

Limitations of our study include the limitations of the Toolset itself, which includes limited ability to assess disparities and some data gaps, such as not including information on non-VA medical care [14]. Since one goal of the project was to use the Toolset as a simpler method of assessing outcomes, we did not gather individual-level outcome data for all participants, limiting our possible analyses. The multi-component nature of the study limited understanding which aspect of the intervention was most effective. Further, the external utility of our findings may vary, especially outside the VA in settings where data toolsets do not already exist, clinical pharmacists with prescriptive authority are less available, or other barriers to use exist, such as higher copayments and more stringent prior authorization requirements. Our results will modestly underestimate the effect, since VAAAHS was a small percentage of overall participants in the control groups. Also, any programs to increase use of these medications elsewhere in VA would make the impact of our intervention appear smaller than it should. We don’t know of any national programs to do that, but some local programs are possible. Finally, we could not distinguish heart failure with preserved ejection fraction from heart failure with reduced injection fraction in the Toolset.

In conclusion, we found that prescription of SGLT2 inhibitors and GLP-1 RAs could be increased substantially with a clinical-level population health intervention and that evaluation could be performed using easy-to-obtain data from existing clinical reports.

## Electronic supplementary material

Below is the link to the electronic supplementary material.

Supplementary Material 1

Supplementary Material 2

## Data Availability

Data is provided within the manuscript or supplementary information files.
