# Supplementary material for: Optimizing SGLT2 inhibitor and GLP-1 RA prescribing in high-risk patients with diabetes: a Department of Veterans Affairs quality improvement intervention
Source: BMC Prim Care. 2025 Mar 21;26:78. doi: 10.1186/s12875-025-02709-0 (PMC11927310; doi:10.1186/s12875-025-02709-0)
Supplement: Supplementary file 1 — Supplementary Material 1 [file 12875_2025_2709_MOESM1_ESM.docx]

**Additional File 1: RE-AIM Framework**

(This is included to present the results in a context appropriate for implementation research.)

| **RE-AIM Dimension** | **Definition** | **Outcome** |
| --- | --- | --- |
| **Reach** | Proportion of patients receiving any intervention | Quantitative:  Number of patients receiving outreach (Figures 2A and 2B), which change over time |
| **Effectiveness** | Change in medication optimization over time | Quantitative:  % of patients with T2DM and CKD, ASCVD, or HF on an SGLT2 inhibitor or GLP-1 RA (Figure 3) |
| **Adoption** | # and % of intervention agents participating (defined as providing outreach outside of scheduled care) | Quantitative:  Home telehealth nurses: (7/7 HT nurses participated) 100% participation  Pharmacists: (13/16 of clinical pharmacist practitioners participated) 81% participation  PCPs: Were not asked to provide outreach outside of scheduled care |
| **Implementation** | Measure of fidelity, burden, and adaptation | Survey of HT nurses and CPPs involved in outreach effort. Post-intervention, participating telehealth nurses and CPPs received a brief 7 question survey. The overall response rate was 50%, including responses from 6 (47%) CPPs and 4 (57%) nurses. Respondents varied in the level of effort with 33% reaching out to greater than 20 patients, 22% reaching out to 11-20 patients, 44% reaching out to 10 or less patients. Respondents spent on average 17 minutes at each outreach encounter. Respondents indicated that the following items facilitated their outreach efforts: education and communication about the purpose of the project and guidelines for qualifying for medication optimization, templates to assist with documentation and review, strong communication among team members, and dashboard data that were easy to use. Barriers to outreach efforts included a lack of time to complete outreach and difficulty reaching patients. |
| **Maintenance** | Proportion of patients receiving medication optimization after the intervention ends | Quantitative:  Prescribing continued to increase faster than at comparison sites (see Figure 3) |
