# Supplementary material for: Optimizing SGLT2 inhibitor and GLP-1 RA prescribing in high-risk patients with diabetes: a Department of Veterans Affairs quality improvement intervention
Source: BMC Prim Care. 2025 Mar 21;26:78. doi: 10.1186/s12875-025-02709-0 (PMC11927310; doi:10.1186/s12875-025-02709-0)
Supplement: Supplementary file 2 — Supplementary Material 2 [file 12875_2025_2709_MOESM2_ESM.pptx]

## Slide 1
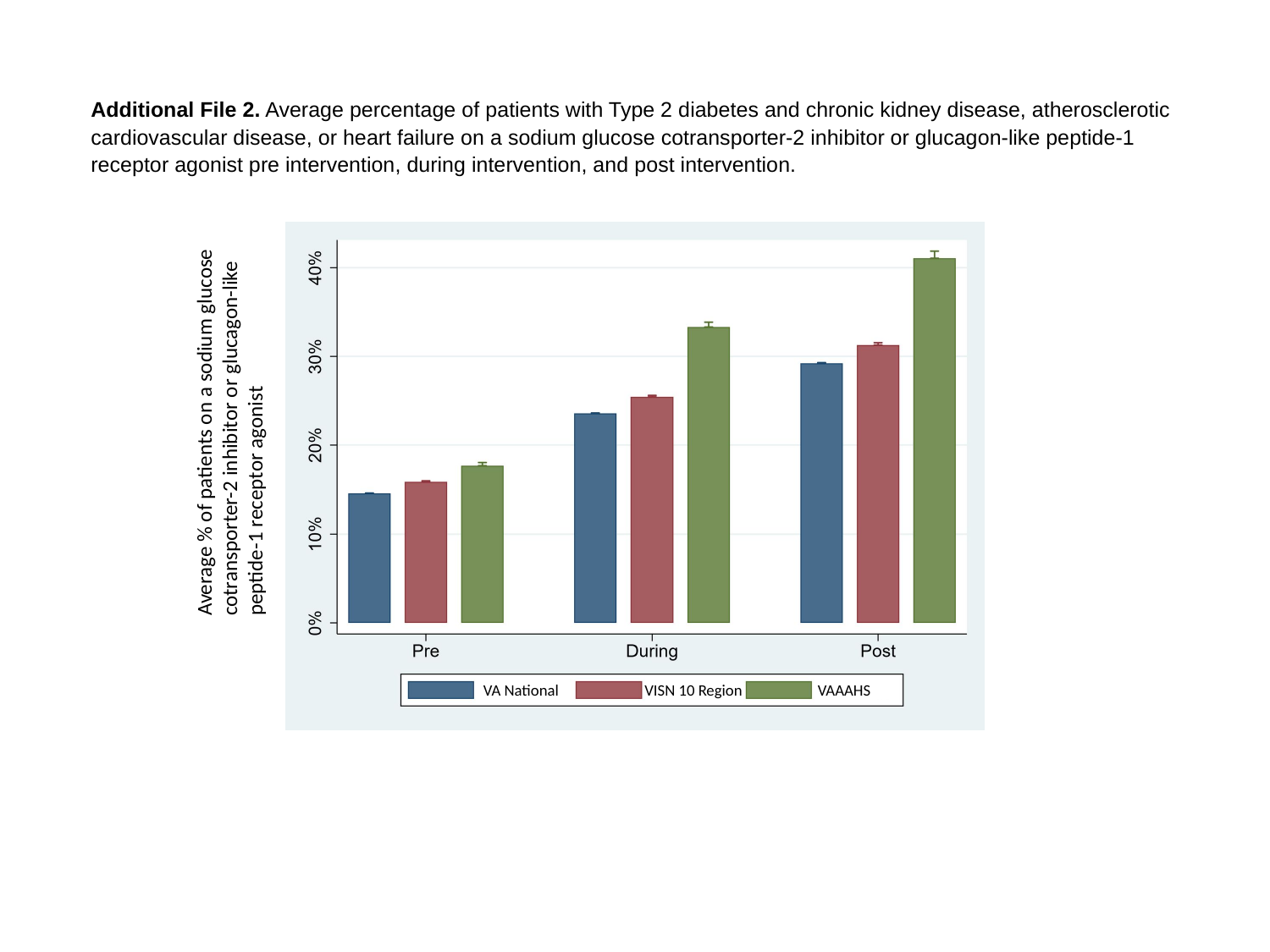

Additional File 2. Average percentage of patients with Type 2 diabetes and chronic kidney disease, atherosclerotic cardiovascular disease, or heart failure on a sodium glucose cotransporter-2 inhibitor or glucagon-like peptide-1 receptor agonist pre intervention, during intervention, and post intervention.
Average % of patients on a sodium glucose cotransporter-2 inhibitor or glucagon-like peptide-1 receptor agonist
VISN 10 Region
VA National
VAAAHS
